# The effectiveness of care manager training in a multidisciplinary plan‐do‐check‐adjust cycle on prevention of undesirable events among residents of geriatric care facilities

**DOI:** 10.1111/ggi.14228

**Published:** 2021-07-07

**Authors:** Shino Ikeda‐Sonoda, Jiro Okochi, Nao Ichihara, Hiroaki Miyata

**Affiliations:** ^1^ Department of Health Policy and Management, Graduate School of Medicine Keio University Tokyo Japan; ^2^ Geriatric Health Services Facility of Tatsumanosato Osaka Japan; ^3^ Department of Home Care Medicine, Graduate School of Medicine University of Tokyo Tokyo Japan; ^4^ Japan Association of Geriatric Health Service Facility Tokyo Japan; ^5^ Department of Healthcare Quality Assessment Graduate School of Medicine, The University of Tokyo Tokyo Japan

**Keywords:** care facilities, fall, geriatric health services, pressure ulcer, risk management

## Abstract

**Aim:**

Undesirable events, such as falls, aspiration, and pressure ulcers, are associated with functional decline and lower quality of life among older adults. This study describes the frequency of such events among residents of geriatric care facilities and assesses the effect of training care managers in a multidisciplinary plan‐do‐check‐adjust cycle on preventing such events.

**Methods:**

This was a Japan‐based, non‐randomized cluster intervention study. The intervention group comprised geriatric care facilities from which care managers had attended a training course, while the control group comprised facilities with care managers who did not receive this training. Six‐month pre‐admission and 3‐month post‐admission incidences of undesirable events were collected from both groups, and the two groups were compared.

**Results:**

Valid data were collected from 862 residents (416 and 446 from the intervention and control groups, respectively) from 130 facilities (60 and 70, respectively). Three‐month post‐admission incidences were 27.8%, 20.0%, and 11.3% for falls, fever, and pressure ulcers, respectively. There was no difference between the groups regarding post‐admission incidence for any event type. Training care managers reduced the post‐admission incidence of pressure ulcers among residents with a history of such ulcers.

**Conclusions:**

The training of care managers in a multidisciplinary risk‐management cycle was not effective for preventing falls, fever, or pressure ulcers. Results underscore the difficulty of preventing risk events in geriatric care facility residents even with organizational training efforts. The authors believe it is important to share such risks with residents and their families. **Geriatr Gerontol Int 2021; 21: 842–848**.

## Background

### Undesirable events

Undesirable events, such as falls, aspiration, and pressure ulcers, can predict functional decline and reduce the quality of life among older adults with disabilities.^1^, ^2^, ^3^, ^4^

Previous studies have examined factors associated with undesirable events,^5^, ^6^, ^7^ and suggested that a history of an undesirable event predicts its recurrence; consequently, simple risk‐assessment tools^8^, ^9^ have been developed using history as a primary predictor. These undesirable events may also be manifestations of frailty syndrome (i.e., collective, aging‐associated decline in multiple physiologic systems).^10^

Residents of geriatric care facilities (GCFs) generally have a high risk of experiencing undesirable events. To our knowledge, no study has examined the frequency of undesirable events among this population; moreover, the influence of organizational factors on this frequency also remains unknown.

### Effectiveness of organizational measures

In GCFs, the staff work to prevent undesirable events by improving the care process and residents' living environments. The frequency of undesirable events represents one of the quality indicators in this setting.^11^, ^12^, ^13^

Certain procedural measures may help to prevent specific undesirable events;^14^ however, the effectiveness of organizational measures for ensuring that frontline care workers consistently implement such procedural measures remains unknown. Many organizations train staff in continuous quality improvement, but the effectiveness of this training is little understood.

**Figure 1:**
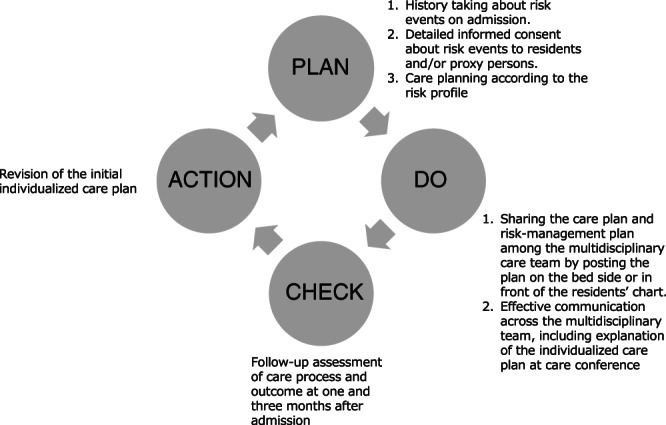
The conceptual framework of the content of the training course. The PDCA cycle, illustrated here, served as the framework for the content of the training course.

The multidisciplinary plan‐do‐check‐adjust (PDCA) cycle represents a potentially effective strategy for care managers, as it may assist in monitoring and improving care, pertains to managing multidisciplinary teams comprising care managers, and can be applied to the prevention of undesirable events (Fig. 1).^15^ In addition, because of their heterogeneous backgrounds, individual care managers may have different levels of skills in risk management. Training on such skills might reduce risk events.

### The role of care managers

As part of Japan's public mandatory long‐term care insurance (LTCI) system, older adults with modest to severe disabilities are allowed to reside in GCFs. Virtually all residents of such facilities use services covered by the LTCI, and these services are coordinated by “care managers.”^17^ Care managers are nationally licensed individuals with at least 5 years' experience as professionals in relevant domains, for example as nurses or social workers, and are responsible for assessing individual needs and planning, coordinating, monitoring, and improving LTCI‐covered services.^15^ They interview and consult residents and discuss care processes with care staff, liaising between in‐person care and administration, and between clinical care and social support. Some care managers also work as members of direct‐care staff and/or as administrators. Guidelines for training care managers^18^ involve facilitating “independence in daily life,” “maintenance of dignity,” and “improved quality of life” for residents. Given the negative impact of undesirable events on these goals, the management of such risks is one of the most effective strategies for care managers.^17^, ^19^ Current training materials for care managers are centered around minimizing the gradual decline in ability of daily life, and the prevention of undesirable events is described only in extraneous detailed discussions. As facility care for disabled elderly persons is complex, skills for continuously improving services should be implemented in care management training.

Care management in clinical settings addresses multiple types of risks, which require various non‐technical skills, including implementing continuous quality improvement in team settings. The multifaceted and continuous nature of care managers' responsibility makes a multidisciplinary PDCA cycle a promising framework for improving their performance. Existing official training materials for care managers describe the importance of continuous *monitoring*, but no formal emphasis is placed on continuous service *improvement* as represented by the PDCA cycle.

This study: (i) describes the incidence of multiple types of undesirable events among newly admitted residents of GCFs in Japan; and (ii) evaluates the effectiveness of a training course for care managers on risk management through a multidisciplinary PDCA cycle in preventing undesirable events among GCF residents.

## Methods

### Participants

**Figure 2:**
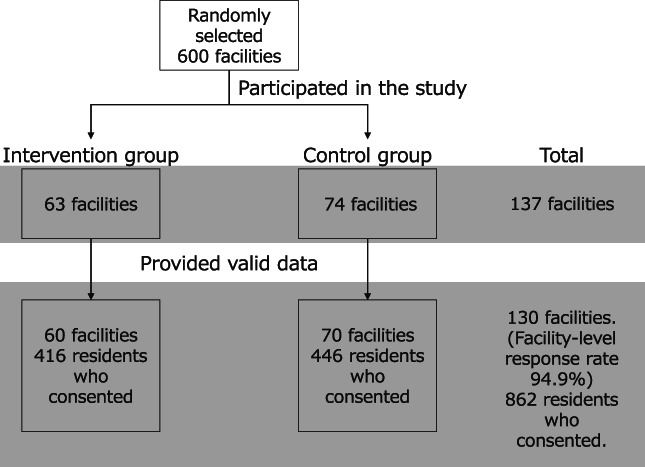
Recruitment and data collection. Of the 600 randomly selected facilities, 137 (63 and 74 in the intervention and control groups, respectively) participated. Of these, 130 (60 and 70 in the intervention and control groups, respectively) provided valid data (facility‐level response rate: 94.9%). Overall, 862 residents of these participating facilities provided informed consent and were analysed.

The Japan Association of Geriatric Health Service Facilities (JAGHSF) has approximately 3600 member facilities, encompassing approximately 90.0% of all GCFs in Japan, all of which operate under the national LTCI scheme.^16^ Of these, 600 facilities were randomly selected and asked to have their care manager(s) attend a 2‐day training course on risk management through a multidisciplinary PDCA cycle. Facilities that opted out were asked to participate in this research as controls. All participating facilities had a standard care‐management process, as stipulated by Japanese LTCI law. For all participating facilities, residents admitted between August and September 2012 (maximum of 10 per facility) received a description of the study and were asked to participate (Fig. 2).

### Intervention

The training course lasted for 2 days and entailed a physical meeting in which the PDCA cycle and multidisciplinary care management were emphasized and other approaches for preventing undesirable events were discussed (Fig. 1). This was part of a risk‐management course run by JAGHSF (4 days in total) and was held 4 weeks prior to enrollment of the participating residents. Figure 1 represents its conceptual framework, and Table S1 summarizes its content. There were seven classes, each taught by an experienced lecturer. Both on‐screen slideshows and printed materials were used. A panel discussion and two group‐work sessions on fall prevention and care management were included. During the course, the importance of the history of specific types of risk events in understanding the risk profile of elderly persons was emphasized.

The study protocol was explained at the end of the course. When outcomes were reported, we confirmed that each facility followed the process described in the training session (data not shown).

### Data collection

All the data were collected using paper forms. Data for participating residents were collected on admission by care managers or social workers during the intake process. Along with age and sex, the required care level was recorded as a proxy measure for the individual's need for care. Required care level is used in the national LTCI system,^16^ and is categorized as 1 (estimated total care <50 min per day), 2 (<70), 3 (<90), 4 (<110), or 5 (≥110).^19^ The participant's activities of daily living and social participation were assessed using the ICF (International Classification of Functioning, Disability and Health) Stages.^20^, ^21^, ^22^ Six‐month pre‐admission histories of undesirable events (i.e., falls, fractures, aspiration, pressure ulcers, dehydration, fever, and infection) were collected. Social participation, functioning, and history of undesirable events were determined through interviewing the residents and/or proxy family members.

The incidence of undesirable events was monitored and recorded regularly for 3 months after admission using existing incidence‐reporting systems. Reported incidences, for which the accuracy was confirmed by the care manager by referring to the care record, were counted as post‐admission incidences. The definitions of each type of incidence are provided in Table S2.

### Analysis

The intervention and control groups were compared regarding residents' characteristics (i.e., age, sex, required care level, functioning, and social participation) and pre‐admission history for each type of undesirable event. The Mann–Whitney U test was used for age (continuous variable) and ordinal categorical variables, and Pearson's chi‐square test was used for non‐ordinal categorical variables.

The effect of the intervention was estimated both as unadjusted odds ratios (ORs) and as ORs adjusted for possible confounders measured. For calculating adjusted ORs, all the variables representing residents' characteristics and pre‐admission history of undesirable events were used as covariates in the multivariable logistic regression models. Pearson's chi‐square test was used to estimate *P‐*values associated with the unadjusted ORs. Absolute risk reduction (ARR) and number needed to treat (NNT), both of which pertain to the point estimates of unadjusted effects of the intervention, were estimated for each event type. A similar analysis was conducted in subgroups of residents with 6‐month pre‐admission histories of same‐type events.

Among all the variables used in the analysis, the following had missing values: age, required care level, and ICF Staging. For multivariable logistic regression, which requires these variables for adjustment, multiple imputation by chained equations was employed, with 10 imputed samples.

R version 4.0.3 (R Core Team, Austria) and its package “micemd”^23^ version 1.6.0, which in turn called “mice”^24^ version 3.13.0, were used for multivariable logistic regression with multiple imputation. JMP® version 14.3 (SAS Institute Inc., USA) was used for (unadjusted) comparison between two groups. A value of *P* < 0.05 was considered to indicate statistical significance. Assuming that the true effect of the intervention was not extremely strong, estimated ORs, unadjusted and adjusted, were presented only when the confidence intervals were within 0.01–100, and otherwise presented as “NA.” No corrections for multiple comparisons were applied in the statistical tests.

Written consent to participate in this study was obtained from each participant, or from the participant's proxy family member if the resident was unable to express their judgment owing to cognitive impairment. This study was approved by the Ethical Review Board of JAGHSF (approval number 23, 1 Aug, 2014) and complies with the Declaration of Helsinki.

## Results

Overall, 862 residents of 130 facilities were included in the analysis (Fig. 2). Table 1 summarizes the residents' characteristics on admission. Of the 862 residents, 263 (30.5%) were men; the median age was 85 years (interquartile range 80–90).

**Table 1:** Residents' characteristics upon admission

		Total (*n* = 862)	Intervention group (*n* = 416)	Control group (*n* = 446)	*P*‐value
Men (%)		599 (69.5%)	288 (69.2%)	311 (69.7%)	0.87
Age		85 (80–90)	85 (79–89)	85 (80–90)	0.31
Required care level	Care level 1	109 (13.1%)	59 (14.8%)	50 (11.5%)	0.06
	Care level 2	173 (20.8%)	79 (19.9%)	94 (21.6%)	
	Care level 3	218 (26.2%)	107 (26.9%)	111 (25.5%)	
	Care level 4	209 (25.1%)	85 (21.4%)	124 (28.5%)	
	Care level 5	124 (14.9%)	68 (17.1%)	56 (12.9%)	
Activity of daily living	1) Basic posture control	4(2–4)	4(2–4)	4(2–4)	0.62
	2) Walking and moving	2(1–2)	2(1–2)	2(1–2)	0.43
	3) Orientation	3(3–4)	3(3–4)	3(3–4)	0.71
	4) Communication	3(2–4)	3(2–4)	3(2–4)	0.94
	5) Mental activities	3(2–4)	3(2–4)	3(2–4)	0.70
	6) Swallowing	4(3–5)	4(3–5)	4(3–5)	0.93
	7) Feeding and feeding assistance	4(4–5)	4(4–5)	4(4–5)	0.79
	8) Toileting	3(2–4)	3(2–4)	3(2–4)	0.02
	9) Bathing	3(2–3)	3(2–3)	3(2–3)	0.90
	10) Oral care	3(2–4)	3(2–4)	3(2–4)	0.96
	11) Self care	3(2–4)	3(2–4)	3(2–4)	0.88
	12) Attaching and detaching clothes	3(2–4)	3(2–4)	3(2–4)	0.53
Social participation	13) Leisure	3(2–3)	3(2–3)	3(2–3)	0.09
	14) Socializing	2(2–3)	2(2–3)	2(2–3)	0.03
Pre‐admission history of undesirable events	Fall	281 (32.6%)	149 (35.8%)	132 (29.6%)	0.05
	Fracture	147 (17.1%)	83 (20.0%)	64 (14.3%)	0.03
	Aspiration	51 (5.9%)	22 (5.3%)	29 (6.5%)	0.45
	Pressure ulcer	93 (10.8%)	44 (10.6%)	49 (11.0%)	0.85
	Dehydration	66 (7.7%)	33 (7.9%)	33 (7.4%)	0.77
	Fever	172 (20.0%)	82 (19.7%)	90 (20.2%)	0.86
	Infection	66 (7.7%)	26 (6.3%)	40 (9.0%)	0.13

Characteristics of residents upon admission. Age and ICF Staging scores (activities of daily living and social participation) are presented as median and interquartile range. Pre‐admission history of undesirable events was defined as the history of undesirable events within the last 6 months before admission.

While the median and interquartile ranges of toileting and socializing scores were the same between the two groups, the score for toileting was worse (more dependent) and that for socializing was better in the intervention group.

The number of missing values in these variables is presented in Table S3.

Among the overall cohort, the frequencies of post‐admission undesirable events were 24.9% (falls), 1.2% (fractures), 2.8% (aspiration), 9.5% (pressure ulcers), 1.0% (dehydration), 17.2% (fever), and 6.2% (infection) (Table 2). On all types of post‐admission events, both with and without adjustment for measured potential confounders, tests on the estimated effect of the intervention did not reject the null hypothesis (*P* > 0.05); that is, the confidence intervals (CIs) of all estimated unadjusted and adjusted ORs included 1.00.

**Table 2:** Frequency of post‐admission undesirable events

Undesirable event	Total (*n* = 862)	Intervention (*n* = 416)	Control (*n* = 446)	Unadjusted OR (95% CI)	*P*‐value	Adjusted OR (95% CI)	*P*‐value	ARR	NNT
Fall	215 (24.9%)	104 (25.0%)	111 (24.9%)	1.01 (0.73–1.39)	0.97	0.25 (0.03–2.43)	0.24		
Fracture	10 (1.2%)	4 (1.0%)	6 (1.4%)	0.71 (0.15–3.03)	0.60	0.25 (0.03–2.43)	0.24	<0.01	>100 †
Aspiration	24 (2.8%)	9 (2.2%)	15 (3.4%)	0.64 (0.24–1.57)	0.28	0.47 (0.16–1.32)	0.15	0.01	73.5
Pressure ulcer	82 (9.5%)	39 (9.9%)	43 (9.6%)	0.97 (0.60–1.57)	0.89	0.95 (0.56–1.59)	0.83	<0.01	>100
Dehydration	9 (1.0%)	4 (1.0%)	5 (1.1%)	0.86 (0.17–4.01)	0.82	0.39 (0.06–2.50)	0.32	<0.01	>100
Fever	148 (17.2%)	62 (14.9%)	86 (19.3%)	0.73 (0.50–1.06)	0.09	0.68 (0.46–1.02)	0.06	0.04	24
Infection	53 (6.2%)	23 (5.5%)	30 (6.2%)	0.81 (0.44–1.47)	0.46	0.82 (0.44–1.51)	0.52	0.01	90.5

Number and proportion of residents who had post‐admission events of each type.

ARR, absolute risk reduction; CI, confidence interval; NNT, number needed to treat; OR, odds ratio.

^†^ ARR and NNT are not presented because of an estimated negative effect.

Similar secondary analyses were conducted on the subgroups of residents with pre‐admission history of the events of the same type (Table 3). In these subgroups without risk adjustment, a significant effect was estimated for pressure ulcers only (15.9% and 38.8% in the intervention and control groups, respectively; unadjusted OR = 0.30, CI = 0.09–0.88, *P* = 0.01). Similarly, adjusted ORs were estimated in fall, pressure ulcer, and fever. (In the other event types, estimation was unstable and their CIs were not within 0.01–100.) No significant effect was observed in these event types, while a trend similar to the unadjusted OR was observed in pressure ulcer (adjusted OR = 0.10, CI = 0.01–1.11, *P* = 0.07).

**Table 3:** Frequency of post‐admission undesirable events among residents with a history of similar pre‐admission events

Undesirable event					Unadjusted OR (95% CI)	*P*‐value	Adjusted OR (95% CI)	*P*‐value	ARR	NNT
	Intervention	Control	Intervention	Control						
Fall	136	123	59 (39.6%)	55 (41.7%)	1.52 (0.55–1.52)	0.72	0.97 (0.56–1.67)	0.91	0.02	47.6
Fracture	77	57	2 (2.4%)	0 (0.0%)	NA ^†^		NA ^†^			^‡^
Aspiration	18	24	2 (9.1%)	6 (20.7%)	0.39 (0.03–2.51)	0.26	NA ^†^		0.14	7.2
Pressure ulcer	37	47	7 (15.9%)	19 (38.8%)	0.30 (0.09–0.88)	0.01	0.10 (0.01–1.11)	0.07	0.22	4.6
Dehydration	32	29	0 (0.0%)	1 (3.0%)	NA ^†^		NA ^†^		0.03	29
Fever	69	83	28 (34.2%)	32 (35.6%)	0.94 (0.48–1.85)	0.80	0.78 (0.34–1.82)	0.57	0.02	71.4
Infection	17	36	5 (19.2%)	5 (12.5%)	1.65 (0.34–8.13)	0.46	NA ^†^			^‡^

Subgroup analysis. For each type of undesirable event, among residents with pre‐admission history of events of the specific type, the proportion of residents who had post‐admission events was compared between the intervention and control groups.

ARR, absolute risk reduction; CI, confidence interval; NNT, number needed to treat; OR, odds ratio.

^†^ ORs, unadjusted or adjusted, are not presented because of imprecise estimation (confidence interval out of 0.01–100 range).

^‡^ ARR and NNT are not presented because of an estimated negative effect.

## Discussion

To our knowledge, this is the first study reporting the incidence of undesirable events among newly admitted GCF residents (Table 2). This is also the first report regarding the effectiveness, in relation to the incidence of undesirable events among newly admitted GCF residents, of training care managers in risk management through multidisciplinary PDCA cycles (Table 2). No significant effect of the intervention was estimated regarding post‐admission incidence of undesirable events, with or without adjusting for measured potential confounders.

As a secondary analysis, the effect of the intervention was estimated in subsets of residents with pre‐admission history of events of the same type (Table 3). Without risk adjustment, a significant effect was estimated for pressure ulcer; but with risk adjustment, no significant effect was observed.

The intervention with the PDCA cycle in this study was not proven effective because (i) intrinsic factors of elderly persons, such as frailty syndrome, play a stronger role than the preventive effects of the intervention, and therefore the effect did not reach statistical significance, and (ii) many of the GCFs had other risk‐management programs with or without PDCA cycles already in place, and the additional intervention in this study could not show a further decrease of undesirable events.

This study differs from previous investigations of measures for preventing undesirable events among GCF residents^14^, ^25^, ^26^, ^27^, ^28^, ^29^ in two major respects. First, this study applied organizational measures that directly targeted care managers as opposed to frontline care staff. Second, this study's intervention emphasized a risk‐management‐focused, multidisciplinary PDCA cycle by examining the frequency of multiple types of undesirable events, which collectively covered the major health‐related undesirable events experienced by GCF residents.

In contrast to our results, some previously tested organizational quality‐improvement programs for preventing specific undesirable events have been shown to be effective. For example, a meta‐analysis by Chang *et al*. showed an OR of 0.82 and an NNT of 11 for a multidisciplinary fall‐prevention program.^30^ In our study, however, no significant difference was observed between the intervention and control groups regarding any type of undesirable event (Table 2). This may be partly because of the generic and long‐term nature of the care managers' roles and the course content, compared with the more specific organizational programs implemented in the reports examined in the meta‐analysis.

### Practical implications

The results of this study can provide evidence‐based information to older individuals and their family members regarding the risks of undesirable events among residents of GCFs. As these risk types are not completely avoidable for frail older individuals, even with high‐quality care, we believe that the communication of such risks is essential. Training on effective communication about such risk events might be beneficial for care providers, residents, and their families. The practical importance of pre‐admission events is discussed in Supporting Information Text S1. The absence of an immediate observed benefit for residents does not preclude the importance of either care management or training of the personnel involved. Without end‐to‐end coordination, monitoring, and quality improvement orchestrated by experts who have direct contact with both residents and care staff, many residents could receive disjointed, low‐quality care, and consequently suffer a higher risk of experiencing undesirable events. Our results also indicate that empowering care managers to reduce some types of risk events, such as pressure ulcers, can potentially benefit high‐risk residents (Table 3) through effective allocation of resources and direction to other professional caregivers, even though the degree of its effectiveness, if indeed a benefit exists, is unknown.

The risk factors for pressure ulcers encompass intrinsic factors such as malnutrition, disease, sensory loss and low mobility, and extrinsic factors such as shear, moisture and friction. In GCFs, nurses, doctors and dieticians primarily intervene to address intrinsic factors. Nurses and care‐workers mainly intervene to alleviate extrinsic factors. Care managers coordinate this role diversity. This study indicates that organizational measures to promote such multidisciplinary interventions might be effective in preventing pressure ulcers; however, understanding the limitations of the PDCA cycle and such training is also necessary.

### Limitations

Our study has several limitations. First, there might be unobserved differences between the groups, for example in staffing, physical care environment, and family support. Second, we did not collect any information about the detailed work process at GCFs. Using checklists might facilitate the work of caregivers who implement specific care processes and collect data on such processes. Third, the observation period of 3 months may have limited the opportunity for detecting certain undesirable events, such as pressure ulcers. Fourth, recall bias on admission may have influenced the result; however, the relatively short 6‐month history period and direct interviews with the residents and/or proxy family members may have helped to decrease the impact of this bias. Fifth, the limited number of observations may have affected the estimation of the intervention's effect, especially in the subgroup analysis. Possible signs of insufficient observations included failure to estimate ORs. A detailed discussion on limitations, including statistical limitations, is presented in Supporting Information Text S1.

### Future research

This study underscores the importance of additional research in multiple ways. The first area that requires further research is the frequency of undesirable events. As in epidemiology, the frequency and associated risks of undesirable events in elderly populations need to be elucidated in various countries and diverse settings of elderly care, such as home‐based care.

The second area concerns organizational measures for improving outcomes of elderly care. This study incorporated multiple aspects of geriatric care, many of which have not been widely investigated – namely, the role of care management, generic continuous quality improvement strategies, and risk events as opposed to gradual functional decline as a target of improvement. A greater focus on high‐risk residents (e.g., those with previous history of risk events), along with a longer period of observation, may allow more opportunities for the identification of effective quality improvement strategies. Collecting information on the cognitive and physiological function of residents, as discussed in previous reports^5^, ^6^, ^7^, may help to remove confounders.

Finally, further investigation is needed regarding the effectiveness of the care staff's communication in helping residents and their families to understand and prepare for the risks of undesirable events and the associated decline in functional status of the elderly.

## Disclosure statement

The authors declare no conflict of interest.

## Consent for publication

Written consent for publication was obtained from the JAGHSF; approval date 1 August 2018.

## Availability of data and materials

All data and materials are preserved by JAGHSF.

## Supporting information

**Table S1** Contents and duration of the training course.

**Table S2** Definition of undesirable events.

**Table S3** Missing values.

**Appendix S1** Supporting Information Text.
