# Supplementary material for: The effectiveness of care manager training in a multidisciplinary plan‐do‐check‐adjust cycle on prevention of undesirable events among residents of geriatric care facilities
Source: Geriatr Gerontol Int. 2021 Jul 7;21(9):842–8. doi: 10.1111/ggi.14228 (PMC8457073; doi:10.1111/ggi.14228)
Supplement: Supplementary file 1 — Table S1 Contents and duration of the training course. [file GGI-21-842-s003.pdf]

(Supporting information) Table 1. Contents and duration of the training course.

	Content	Duration (min)
Lectures	Introduction	60
	Risk management and law	120
	Coaching method	60
	Handling human errors	180
	Complaints handling	60
	Minor accident and occurrence of complaint	60
	Research design and methods	60
Panel discussion and workshop	Panel discussion	120
	Workshop on fall prevention	180
	Workshop on care management	165
