# Supplementary material for: The effectiveness of care manager training in a multidisciplinary plan‐do‐check‐adjust cycle on prevention of undesirable events among residents of geriatric care facilities
Source: Geriatr Gerontol Int. 2021 Jul 7;21(9):842–8. doi: 10.1111/ggi.14228 (PMC8457073; doi:10.1111/ggi.14228)
Supplement: Supplementary file 2 — Table S2 Definition of undesirable events. [file GGI-21-842-s001.pdf]

(Supporting information) Table 2. Definition of undesirable events

Event type	Description
Fall	An event that results in a person coming to rest inadvertently on the ground or floor or other lower level
Fracture	Ranging from cracks in a bone to a bone that is separated and out of place, as diagnosed by physicians using X-ray or other imaging systems
Aspiration	Pneumonia resulting from inhalation of foreign bodies (such as food particles)
Pressure ulcer	Localized damage to the skin and underlying soft tissue, usually over a bony prominence or related to medical or other devices. In this study, pressure injury of greater than stage 1 (non-blanchable erythema of intact skin) was also counted
Dehydration	Diagnosed using common symptoms, including tachycardia (>100 bpm), low systolic blood pressure (<100 mm Hg), dry mucous membrane, dry axilla, poor skin turgor, sunken eyes, long capillary refill time, urine color, and urine specific gravity
Fever	37.5° Celsius, as measured by a common thermometer.
Infection	Infectious disease with known causes, such as tuberculosis, herpes etc.
