# Supplementary material for: The effectiveness of care manager training in a multidisciplinary plan‐do‐check‐adjust cycle on prevention of undesirable events among residents of geriatric care facilities
Source: Geriatr Gerontol Int. 2021 Jul 7;21(9):842–8. doi: 10.1111/ggi.14228 (PMC8457073; doi:10.1111/ggi.14228)
Supplement: Supplementary file 3 — Table S3 Missing values. [file GGI-21-842-s004.pdf]

(Supporting infromation) Table 3. Missing values

Variable	N	%
Sex	0	0.0%
Age	10	1.2%
Required care level	29	3.4%
ICF staging level on admission (any item)	14	1.6%

Numbers and proportion of missing values in pre-admission variables.
Distribution of observed values of these variables are presented in Table 1.
