# Supplementary material for: The effectiveness of care manager training in a multidisciplinary plan‐do‐check‐adjust cycle on prevention of undesirable events among residents of geriatric care facilities
Source: Geriatr Gerontol Int. 2021 Jul 7;21(9):842–8. doi: 10.1111/ggi.14228 (PMC8457073; doi:10.1111/ggi.14228)
Supplement: Supplementary file 4 — Appendix S1 Supporting Information Text. [file GGI-21-842-s002.pdf]

Supporting Information Text

Importance of pre-admission history in identifying high-risk residents

Results of the current study highlight that determining pre-admission incidence is a simple method of identifying residents at a high risk of experiencing specific types of undesirable events. If event history is adequately collected by GCFs and shared among care staff, a three-step process can be implemented to reduce the risk of these undesirable events and consequently improve the quality of care. The first step concerns introducing a standardized risk-management process using the PDCA cycle by collecting information, including pre-admission history of undesirable events, and performing risk-prevention planning and follow-up. The second step concerns educating the care staff regarding the risk-management process and sharing information regarding risk factors. The third step concerns sharing information among the multidisciplinary team and implementing measures to prevent undesirable events.[1]

Detailed discussion of limitations

Possible unobserved differences between groups

There might be unobserved differences in relevant features between the groups, such as number of staff members, level of staff experience, staff morale, physical care environment, and family support to residents. Also, cognitive function and physiological factors, e.g., nutrition, anemia, and heart failure, of the residents may be confounding the results.

Absence of information on detailed care process

We did not collect any information about the detailed work process of either the care managers or the direct care staff. Using checklists might facilitate the work of caregivers who implement specific care processes and collect data on such processes.

Limited observation period

The observation period of three months post-admission may have limited the opportunity for detecting certain undesirable events, such as pressure ulcers.

Possible recall bias on pre-admission history

Recall bias on admission may have influenced the result; however, the relatively short six-month history period and direct interviews with the residents and/or proxy family members may have helped to decrease the impact of this bias.

Limited number of observations

The limited number of observations, in relation to the incidence of undesirable events, may have resulted in insufficient statistical power to estimate effect of the intervention, especially in the subgroup analysis of only residents with pre-admission history of undesirable events. Possible signs of insufficient observations included wide confidence intervals of ORs, and failure to estimate ORs in the subgroup analysis

Statistical limitations

Increased possibility of type-I errors due to multiple comparison

The overall possibility of type-I errors (i.e., dictating non-existing correlations) might be higher than the nominal significance threshold (0.05) due to the multiple comparisons performed.

Model dependency

Model dependency inherent to regression adjustment might have caused misleading results. Alternative approaches that are free from modeling assumptions include matching and weighting.

Hierarchical nature of data

The methods of comparing results between the intervention and control groups did not address the hierarchical nature of the data (i.e., residents were members of facilities). Generalized estimating equations and mixed models can be employed to address such data structures.

REFERENCES

1. Nicolay CR, Purkayastha S, Greenhalgh A, Benn J, Chaturvedi S, Phillips N, et al. Systematic review of the application of quality improvement methodologies from the manufacturing industry to surgical healthcare. British Journal of Surgery. 2012.
